# Renal vascular lesions in IgA nephropathy

**DOI:** 10.12861/jrip.2013.14

**Published:** 2013-06-01

**Authors:** Azar Baradaran

**Affiliations:** ^1^Department of Pathology, Isfahan University of Medical Sciences, Isfahan, Iran

**Keywords:** Immunoglobulin A nephropathy, Oxford classification, Vasculopathy, Thrombotic microangiopathy

**Implication for health policy/practice/research/medical education:**

The significance of arterial lesions in IgAN patients has not been investigated well and characteristics of renal vessels in this disease not been studied separately. More investigations is necessary to found the clinical significance of thrombotic microangiopathy in IgAN.

Vascular lesions are of principal importance in the progression of various primary and secondary kidney diseases (1). In the majority of individuals with kidney diseases, vascular component, is involved secondarily in the disease process affecting primarily the glomeruli. Indeed, in IgAN as a common and progressive glomerulopathy, impaired kidney function, hypertension, proteinuria and interstitial fibrosis are the greatest and most reliable predictors of poor outcome in IgAN (1,2). However, the significance of arterial lesions in IgAN patients has not been investigated well and characteristics of renal vessels in this disease not been studied separately. After the publication of Oxford classification of IgAN, and including the four morphologic lesions of mesangial proliferation, endocapillary proliferation, mesangial sclerosis and final interstitial fibrosis/tubular atrophy to this classification (3,4), the clinical significance of other morphologic lesions such as fibrinoid necrosis of capillary walls, thrombotic microangiopathy (TMA) had not yet been clarified. Previously, El Karoui *et al*. in a study on a group of IgAN patients found 53% patients had morphologic lesions of TMA (5). They suggested that, morphologic lesions of TMA are prevalent in IgAN, however, this result was in contrast to the study of Nasri *et al*. They found the morphologic lesions of TMA in 1.4% of their patient (6). Hence, more investigations is necessary to found the clinical significance of TMA in IgAN.

## Author’s contribution

AB is the single author of the manuscript.

## Conflict of interests

The authors declared no competing interests.

## Ethical considerations

Ethical issues (including plagiarism, misconduct, data fabrication, falsification, double publication or submission, redundancy) have been completely observed by the authors.

## Funding/Support

None.
